# Social and demographic factors associated with sports nutrition knowledge and food habits among university athletes

**DOI:** 10.3389/fnut.2026.1838485

**Published:** 2026-05-29

**Authors:** Adam Tawfiq Amawi, Walaa Jumah Alkasasbeh, Hebah Abdalla Ali, Bekir Erhan Orhan

**Affiliations:** 1 Department of Movement Sciences and Sports Training, School of Sports Sciences, The University of Jordan, Amman, Jordan; 2 Department of Physical Education, School of Sports Sciences, The University of Jordan, Amman, Jordan; 3 Department of Nutrition and Food Technology, School of Agriculture, The University of Jordan, Amman, Jordan; 4 Faculty of Sports Sciences, Istanbul Aydin University, Istanbul, Türkiye

**Keywords:** food habits, health promotion, healthy lifestyle, nutrition education, public health, sports nutrition knowledge, university athletes

## Abstract

**Background:**

Sports nutrition knowledge (SNK) and food habits (FH) among university athletes may be influenced by various social, demographic, and training-related factors. However, evidence regarding how these factors relate to SNK and FH remains limited and inconsistent in university sport settings.

**Objective:**

This study aimed to examine the social and demographic factors associated with SNK and FH among university athletes and to investigate the relationship between SNK and FH.

**Methods:**

A cross-sectional study was conducted among 89 university athletes who completed validated questionnaires assessing SNK and FH. Descriptive statistics were calculated, and Pearson’s correlation coefficient, independent *t*-tests, and one-way ANOVA were used to examine associations and group differences according to gender, academic year, training experience, and body mass index (BMI).

**Results:**

A small but statistically significant positive correlation was found between SNK and FH (*r* = 0.21, *p* = 0.047), indicating that higher nutrition knowledge was associated with healthier dietary habits. Significant differences in SNK were observed according to academic year (*p* < 0.001) and training experience (*p* = 0.027), while FH differed significantly according to academic year (*p* = 0.014). No significant differences were found according to gender or BMI. Overall, participants demonstrated low levels of SNK and moderate levels of FH. The findings also suggest that social and environmental influences may contribute to shaping dietary behaviors beyond nutrition knowledge alone.

**Conclusion:**

Social, demographic, and training-related factors appear to play a role in shaping SNK and FH among university athletes. Although SNK was modestly associated with healthier food habits, knowledge alone may not be sufficient to promote optimal dietary behaviors. These findings highlight the importance of structured nutrition education programs that address both nutrition knowledge and the broader social and practical factors influencing healthy eating within university sport environments.

## Introduction

Nutrition is essential for athletic performance, recovery, and long-term health (1). Compared to the general population, athletes require greater energy and nutrient intake to sustain training, support muscle adaptation, and protect immune function (1, 2). Adequate nutrition improves endurance, strength, agility, and cognitive performance, which are critical for student-athletes managing both academics and sport (3–5). In contrast, poor dietary intake can cause fatigue, slower recovery, illness, and higher injury risk (6, 7). These outcomes emphasise the need for consistent and effective nutritional practices among university athletes.

Sports nutrition knowledge (SNK) is a key factor shaping athletes’ dietary behavior, covering macronutrients, micronutrients, hydration, supplementation, and meal timing (8–10). Athletes with higher SNK may be more likely to adopt healthier FH to meet sport-specific demands (11). Evidence remains mixed: while some studies show positive links between knowledge and diet, others report weak or inconsistent results (4, 12, 13). This suggests that behavior depends not only on knowledge but also on psychosocial and structural factors such as cost, access, and food availability (14, 15). For university athletes, independence often means taking charge of meals, but financial limits, demanding schedules, and limited healthy dining options make applying nutrition knowledge difficult (9, 16–18).

Demographic and training-related factors also influence both SNK and FH (19). Academic year progression often brings greater exposure to sport science education and experience, meaning senior students may demonstrate higher knowledge than newer students (20, 21). Training experience can also instill discipline and reinforce healthier eating patterns (1, 22). Similarly, BMI shapes nutrition-related behaviors: athletes with normal BMI typically show healthier practices, while those who are overweight or obese may struggle due to environmental or knowledge-related barriers (9, 23, 24).

Despite the recognized importance of nutrition in athletic contexts, gaps remain in understanding the relationship between SNK and FH among university athletes, particularly when considering the combined influence of academic year, training experience, and BMI. Previous studies have frequently assessed nutrition knowledge or dietary practices in isolation, or across broad athletic populations, without examining how these intersecting variables may shape the knowledge–behavior dynamic (10, 18, 25). Although international research often reports low SNK among athletes, evidence focused on university settings where students balance academic and athletic demands remains limited (9, 18). Closing this gap is crucial for designing evidence-based interventions, education, and policies that strengthen both nutrition knowledge and its practical application among student-athletes (26).

The exact manner in which these variables influence the association between SNK and FH is not thoroughly explored, with some studies presenting contradictory or inconsistent results. We aimed to assess the level of SNK and FH among university athletes, as well as to explore the relationship between SNK and their FH. Additionally, the study aimed to determine whether the levels of SNK and FH differed according to several demographic and training-related variables, including gender, academic year, training experience, and BMI. This study hypothesized a significant positive relationship between SNK and FH among university athletes. Additionally, it was hypothesized that the levels of SNK and FH would differ according to several demographic and training-related variables, including gender, academic year, training experience, and BMI.

## Materials and methods

### Participants

A total of 89 university athletes (44 males and 45 females) participated in this cross-sectional study. Participants were recruited using a convenience sampling approach from university sports teams.

Participants were recruited from various sports programs within the university, including both team and individual sports such as football, basketball, athletics, and martial arts, with varying ages and training experience, categorized as 2–4 years, 5–7 years, 8–10 years, and 11 years or more. BMI was calculated and classified into four categories: underweight (<18.5), normal weight (18.5–24.9), overweight (25–29.9), and obese (>30).

Inclusion criteria included university athletes enrolled in regular sports programs with a minimum training experience of 2 years who provided informed consent. Exclusion criteria were injuries preventing training, chronic illnesses affecting nutrition or athletic performance, or lack of informed consent.

Table 1 presents the demographic and training characteristics of the participants (Table 1). A total of 89 university athletes participated in the study, including 44 males (49.4%) and 45 females (50.6%). Regarding academic year, participants were distributed as follows: 23 first-year students (25.8%), 21 second-year students (23.6%), 21 third-year students (23.6%), and 24 fourth-year students (27.0%). In terms of training experience, the majority of participants had 5–7 years of experience (47.2%), followed by 8–10 years (22.5%), 2–4 years (20.2%), and 11 years or more (10.1%). The distribution of BMI showed that 47.2% of the athletes were classified as underweight, 34.8% had normal weight, 14.6% were overweight, and 3.4% were classified as obese. The relatively high proportion of athletes classified as underweight may be explained by the diversity of sports represented in the sample, including sports that are typically associated with lower body mass indices and lean body composition. In addition, the use of self-reported height and weight measures may have contributed to some degree of measurement variability.

**Table 1:** Demographic characteristics of participants.

Variables		Number	Percentage %
Gender	Male	44	49.4
	Female	45	50.6
Academic year	First year	23	25.8
	Second year	21	23.6
	Third year	21	23.6
	Fourth year	24	27.0
Training experience	2–4 years	18	20.2
	5–7 years	42	47.2
	8–10 years	20	22.5
	11 years and more	9	10.1
BMI	<18.5 kg/m^2^	42	47.2
	18.5–24.9 kg/m^2^	31	34.8
	25–29.9 kg/m^2^	13	14.6
	>30 kg/m^2^	3	3.4
Total		89	100

### Procedures

The study was conducted using a cross-sectional design. Prior to data collection, participants were provided with a detailed explanation of the study’s objectives, procedures, and their rights as participants, and written informed consent was obtained from all participants. Data were collected using a structured questionnaire consisting of two main sections: SNK and FH. Participants completed the questionnaire individually in a quiet setting and were given sufficient time to answer all items. The SNK section consisted of 59 items assessing knowledge of macronutrients, micronutrients, hydration, and frequency of food intake, with correct answers scored as +1, incorrect answers as −1, and unanswered items as 0. The FH section consisted of 13 items evaluating dietary practices, scored on a 0–3 scale, with higher scores indicating healthier habits. Participants’ height and weight data were used to calculate BMI in kilograms per square meter (kg/m^2^). Previous studies have shown a strong correlation between self-reported BMI and measured BMI (27). The reported height and weight data were then compared with the CDC growth charts to identify any extreme deviations from self-reported values (28). All questionnaires were reviewed for completeness, and any missing or unclear responses were clarified with the participants. Data collection was conducted in accordance with ethical guidelines, ensuring confidentiality and voluntary participation throughout the process. The study was approved by the Institutional Review Board (IRB) of Al-Ahliyya Amman University, Amman, Jordan (Approval No. AAU-IRB-2024/0211).

### Instruments

Two validated instruments were used in this study to assess SNK and FH among the participants.

### Sports nutrition knowledge (SNK)

SNK was measured using the SNK for Athletes Survey, a well-established and validated tool developed specifically for athletes (29). The survey consisted of 59 items covering a wide range of domains, including knowledge of macronutrients, micronutrients, hydration strategies, dietary supplements, and meal frequency. Participants received +1 point for each correct response, −1 point for an incorrect response, and 0 points if the item was left unanswered. This scoring approach was adopted from the original validated questionnaire and was designed to reduce the likelihood of guessing by penalizing incorrect responses while distinguishing unanswered items from incorrect answers. The total possible score ranged from 0 to 59, with higher values indicating greater nutritional knowledge.

### Food habits (FH)

Dietary behaviors were assessed using a 13-item FH questionnaire (30). The original version contained 14 items; however, one question regarding the consumption of alcoholic beverages during meals was excluded, as it was not culturally relevant to the target population. The items focused on daily eating practices such as breakfast consumption, number of meals per day, intake of fruits and vegetables, and consumption of sugary or carbonated drinks. Seven of the questions used a four-point Likert scale (“always,” “often,” “sometimes,” “never”), while the remaining six items employed alternative four-option formats. Responses were scored on a 0–3 scale, where higher scores reflected healthier dietary habits. The cumulative score ranged from 0 to 39.

### Psychometric properties of the instruments

The internal consistency of the study instruments was assessed using Cronbach’s alpha. The SNK questionnaire demonstrated acceptable reliability in the current sample (Cronbach’s *α* = 0.80). Similarly, the adapted FH questionnaire showed acceptable internal consistency after the cultural modification (Cronbach’s *α* = 0.70). The modification of the FH questionnaire was limited to the removal of one alcohol-related item due to cultural irrelevance and did not substantially alter the overall structure or content of the instrument. However, additional psychometric evaluation, including construct validity testing, is recommended in future studies. These findings indicate that both instruments were reliable for assessing sports nutrition knowledge and dietary behaviors among university athletes.

### Statistical analysis

The statistical analysis was conducted using SPSS version 16.0. Prior to conducting parametric analyses, assumptions of normality and homogeneity of variance were examined using the Shapiro–Wilk test and Levene’s test, respectively. The assumptions were considered adequately satisfied for the applied analyses. Descriptive statistics were calculated, and Pearson’s correlation coefficient was used to examine the association between sports nutrition knowledge and food habits. Independent sample *t*-tests were used to compare SNK and FH scores between male and female athletes, while one-way ANOVA was conducted to examine differences according to academic year, training experience, and BMI categories. A *post-hoc* power analysis indicated that the achieved sample size was adequate to detect small-to-moderate effect sizes at *α* = 0.05.

## Results

This study hypothesized a significant positive relationship between SNK and FH among university athletes and that both SNK and FH would differ according to demographic and training-related variables, including gender, academic year, training experience, and BMI. The results are presented in line with these hypotheses. First, the correlation analysis examined the association between SNK and FH. Next, independent-sample *t*-tests and one-way ANOVA were used to determine whether differences existed in SNK and FH across gender, academic year, training experience, and BMI.

Pearson’s correlation analysis revealed a small but statistically significant positive association between SNK and FH (*r* = 0.21, *p* = 0.047, 95% CI 0.01–0.39) (see Table 2). This finding indicates that athletes with higher levels of sports nutrition knowledge tended to report healthier food habits. Although the strength of the correlation was relatively weak, the positive direction of the relationship suggests that improved nutrition knowledge may contribute to better dietary behaviors among university athletes. The confidence interval did not cross zero, further supporting the statistical significance of the observed association. However, the modest correlation coefficient also indicates that factors other than nutrition knowledge are likely involved in shaping food habits among athletes.

**Table 2:** Pearson correlation between sports nutrition knowledge (SNK) and food habits (FH).

*N*	*r*	*p*-value	95% CI
89	0.21	0.047	0.01–0.39

Table 3 presents the levels of SNK and FH among university athletes. The mean SNK score was 12.60 ± 10.97 out of a possible 59, with scores ranging from 0 to 59. Based on the scoring classification, the level of sports nutrition knowledge among the participants was considered low. For FH, the mean score was 25.47 ± 5.79 out of a possible 39, with the scale ranging from 0 to 39. According to the scoring classification, the level of food habits among the university athletes was considered moderate.

**Table 3:** Levels of SNK and FH among university athletes.

Variables	*N*	Mean ± SD	Scale range	Level
SNK	89	12.60 ± 10.97	0–59	Low
FH	89	25.47 ± 5.79	0–39	Moderate

SNK, sports nutrition knowledge; FH, food habits.

Table 4 presents the differences in SNK and FH scores between male and female university athletes. For SNK, male athletes demonstrated a higher mean score (14.23 ± 8.76) compared with female athletes (11.00 ± 12.67). However, the independent samples *t*-test showed that this difference was not statistically significant (*t* = 1.394, *p* = 0.167). Similarly, for FH, male athletes reported a slightly higher mean score (26.14 ± 5.65) compared with female athletes (24.82 ± 5.90). Nevertheless, the difference between males and females was also not statistically significant (*t* = 1.071, *p* = 0.287). Overall, the findings indicate that no significant gender-related differences were observed in either sports nutrition knowledge or food habits among the participants.

**Table 4:** Differences in SNK and FH by gender.

Variables	Gender	*n*	Mean ± SD	df	*t*	*p*-value
SNK	Male	44	14.23 ± 8.76	87	1.394	0.167
	Female	45	11.00 ± 12.67			
FH	Male	44	26.14 ± 5.65	87	1.071	0.287
	Female	45	24.82 ± 5.90			

SNK, sports nutrition knowledge; FH, food habits.

Table 5 presents the differences in SNK and FH scores according to academic year, training experience, and BMI. Significant differences in SNK scores were observed according to academic year (*F* = 9.387, *p* < 0.001) and training experience (*F* = 3.218, *p* = 0.027), whereas no significant differences were identified according to BMI (*p* > 0.05). *Post-hoc* analyses indicated that second-year students differed significantly from first-, third-, and fourth-year students in SNK scores. In addition, athletes with 5–7 years of training experience demonstrated significantly lower SNK scores compared with those with 2–4 years and 8–10 years of training experience.

**Table 5:** SNK and FH scores according to academic year, training experience, and BMI.

Variable	Category	*n*	Mean ± SD	*F*	*p*-value
SNK					
Academic year	First year	23	16.00 ± 6.39	9.387	<0.001
	Second year	21	13.19 ± 12.38		
	Third year	21	13.00 ± 12.53		
	Fourth year	24	17.21 ± 6.11		
	Total	89	12.60 ± 10.98		
Training experience	2–4 years	18	16.28 ± 9.46	3.218	0.027
	5–7 years	42	8.93 ± 11.69		
	8–10 years	20	15.95 ± 8.87		
	11 years and more	9	14.89 ± 10.61		
	Total	89	12.60 ± 10.98		
BMI	<18.5 kg/m^2^	42	11.83 ± 11.10	1.215	0.309
	18.5–24.9 kg/m^2^	31	14.26 ± 10.10		
	25–29.9 kg/m^2^	13	13.46 ± 12.02		
	>30 kg/m^2^	3	2.33 ± 12.66		
	Total	89	12.60 ± 10.98		
FH					
Academic year	First year	23	22.61 ± 6.01	3.724	0.014
	Second year	21	26.76 ± 6.10		
	Third year	21	24.86 ± 5.48		
	Fourth year	24	27.63 ± 4.51		
	Total	89	25.47 ± 5.79		
Training experience	2–4 years	18	23.72 ± 5.27	1.531	0.212
	5–7 years	42	25.76 ± 6.02		
	8–10 years	20	27.25 ± 6.39		
	11 years and more	9	23.67 ± 2.83		
	Total	89	25.47 ± 5.79		
BMI	<18.5 kg/m^2^	42	25.98 ± 7.01	2.152	0.100
	18.5–24.9 kg/m^2^	31	26.42 ± 4.65		
	25–29.9 kg/m^2^	13	22.69 ± 2.18		
	>30 kg/m^2^	3	20.67 ± 3.51		
	Total	89	25.47 ± 5.79		

SNK, sports nutrition knowledge; FH, food habits.

For FH, significant differences were observed according to academic year (*F* = 3.724, *p* = 0.014), whereas no significant differences were found according to training experience or BMI (*p* > 0.05). *Post-hoc* analysis revealed a significant difference only between first-year and fourth-year students, with fourth-year students demonstrating healthier food habits.

## Discussion

The present study examined the relationship between SNK and FH among university athletes and explored whether these variables differed according to gender, academic year, training experience, and BMI. The findings revealed a small but statistically significant positive association between SNK and FH, indicating that athletes with higher nutrition knowledge tend to report healthier dietary habits. Although the association was statistically significant, the strength of the relationship was relatively modest, indicating that sports nutrition knowledge explains only a limited proportion of the variance in food habits. This suggests that additional psychological, environmental, and social factors may also play an important role in shaping dietary behaviors among university athletes.

This finding is consistent with previous research suggesting that nutrition knowledge may play an important role in dietary behaviors among athletes (31, 32). Several studies have reported that athletes who possess greater knowledge regarding macronutrient requirements, hydration strategies, and meal timing are better equipped to adopt dietary practices that support training demands and recovery processes (1, 33). However, the strength of the association observed in the current study was relatively modest, which aligns with previous literature showing that knowledge alone may not fully translate into optimal dietary behaviors (34). Behavioral outcomes are influenced by multiple factors, including time constraints, food availability, financial limitations, and social influences that can affect athletes’ ability to apply nutrition knowledge in daily life (35–38). These findings may be interpreted within behavioral frameworks suggesting that nutrition-related behaviors are shaped not only by knowledge, but also by social, environmental, and psychological determinants (39–41).

Despite the positive relationship between SNK and FH, the overall level of sports nutrition knowledge among the participants was low. This result is consistent with findings reported in several international studies indicating that many athletes, including those competing at university level, demonstrate insufficient nutrition knowledge (42, 43). Limited formal nutrition education within sports programs and reliance on informal information sources such as peers, coaches, or social media may contribute to these knowledge gaps. Low nutrition knowledge among athletes is concerning because inadequate dietary practices may negatively influence performance, recovery, immune function, and injury risk (22, 44, 45).

This finding may also reflect the well-documented “knowledge–behavior gap” frequently reported in nutrition research (11, 46). Individuals may demonstrate relatively acceptable dietary behaviors despite limited formal nutrition knowledge due to environmental influences, cultural eating patterns, peer behaviors, or guidance from coaches and teammates (11, 47, 48). Therefore, while nutrition knowledge plays an important role in shaping dietary practices, it represents only one component within a complex set of psychological, social, and environmental factors that influence athletes’ eating behaviors (37, 49).

Interestingly, the results showed no significant differences in SNK or FH between male and female athletes. This finding suggests that gender may not be a major determinant of nutrition knowledge or dietary practices in university sport settings. This may be explained by the fact that both male and female athletes in the current study were exposed to similar training environments, educational experiences, and access to nutrition-related information within the university setting. Previous research among collegiate athletes has reported significant gender differences in nutritional practices and perceptions related to body weight and diet (50). Nevertheless, previous studies have also reported gender differences in dietary behaviors among collegiate athletes, including variations in fast food consumption and meal preparation habits (51).

Academic year emerged as one of the most influential variables in the current study. Athletes in later academic years demonstrated significantly higher SNK and healthier FH compared with those in earlier years. This trend may reflect increased exposure to sport science education, greater training maturity, and accumulated experience in managing both academic and athletic responsibilities. As students progress through university, they may develop stronger awareness of the importance of nutrition in supporting athletic performance and overall health (52). These findings highlight the potential role of educational progression in improving nutrition-related competencies among student-athletes.

Training experience was also associated with differences in sports nutrition knowledge, although it did not significantly influence food habits. Athletes with greater training experience may acquire nutrition-related information through prolonged exposure to coaches, sports staff, and competitive environments (53). However, the lack of a significant association between training experience and FH suggests that experience alone may not be sufficient to modify daily eating behaviors. Practical barriers such as limited time, convenience-based food choices, and access to healthy meals may continue to affect athletes regardless of their training background (19, 37, 54).

The study did not identify significant differences in SNK or FH according to BMI categories. This finding may indicate that body weight status is not necessarily associated with nutrition knowledge or eating behaviors among university athletes (55). Previous research has reported mixed findings regarding the relationship between BMI and nutrition behaviors, with some studies suggesting that athletes with normal BMI demonstrate healthier dietary practices than those with overweight or obesity (56).

Overall, the findings emphasize that although nutrition knowledge is positively related to dietary habits, improving knowledge alone may not be sufficient to ensure optimal nutritional practices among university athletes. From a practical perspective, these findings highlight the importance of implementing structured sports nutrition education within university athletic environments. Integrating nutrition education modules into sport science curricula, providing workshops for athletes, and offering access to qualified sports dietitians may help improve nutrition knowledge and support the translation of knowledge into healthier dietary practices (57, 58). Such initiatives may ultimately contribute to improving both athlete health and sport performance. Workshops, individualized counseling, and collaboration with sports dietitians could help bridge the gap between knowledge and practice. Implementing such interventions early in university athletic programs may enhance athletes’ understanding of sports nutrition and encourage healthier dietary patterns throughout their academic and sporting careers.

## Conclusion

This study examined the social, demographic, and training-related factors associated with sports nutrition knowledge (SNK) and food habits (FH) among university athletes, in addition to investigating the relationship between SNK and FH. The findings revealed a modest but significant positive association between sports nutrition knowledge and dietary habits, indicating that athletes with higher levels of nutrition knowledge tended to demonstrate healthier eating behaviors. Despite this association, the overall level of sports nutrition knowledge among the participants was relatively low, highlighting a potential gap in nutrition education within university athletic environments.

Academic year and training experience were identified as important factors associated with sports nutrition knowledge, while academic year was also associated with food habits, suggesting that greater exposure to academic learning and athletic experience may contribute to improved nutrition awareness and dietary practices. In contrast, no significant differences were observed according to gender or BMI. The findings also suggest that social and environmental influences may play a role in shaping dietary behaviors beyond nutrition knowledge alone.

These findings emphasize the importance of implementing structured and accessible nutrition education programs within university sports settings that address not only nutrition knowledge but also the broader social and practical factors influencing healthy eating behaviors. Such strategies may help bridge the gap between knowledge and practice and support healthier dietary habits and athletic well-being. Future research should consider larger and more diverse samples, longitudinal designs, and the inclusion of additional social, behavioral, and environmental factors that may influence athletes’ nutritional practices and dietary behaviors over time.

### Limitations and strengths

Like any research, this study has several limitations that should be acknowledged when interpreting the findings. First, the cross-sectional design limits the ability to establish causal relationships between SNK and FH. While a significant association was identified, it cannot be concluded whether higher nutrition knowledge directly leads to healthier dietary behaviors or whether athletes with healthier habits seek more nutrition-related information.

In addition, the relatively small sample size and recruitment of participants from a single university using a convenience sampling approach may limit the generalizability of the findings and increase the risk of selection bias. The study also relied on self-reported data for dietary habits, height, and weight, which may introduce reporting bias or inaccuracies despite previous evidence supporting the acceptable validity of self-reported anthropometric measures. Furthermore, the unequal distribution of participants across BMI categories, particularly the very small number of participants classified as obese, may have reduced the statistical power for subgroup comparisons and limited the reliability of BMI-related findings. Another limitation is that the statistical analysis was restricted to bivariate techniques, and no multivariate analyses were conducted to control for potential confounding variables. In addition, several environmental, psychological, and social determinants that may influence the relationship between sports nutrition knowledge and dietary behaviors were not assessed.

Despite these limitations, the present study has several important strengths. One key strength is the use of validated and reliable instruments to assess both SNK and dietary habits, ensuring methodological rigor and measurement consistency. The study also contributes to the growing body of literature by simultaneously examining the relationship between SNK and FH while considering multiple demographic and training-related variables, including academic year, training experience, gender, and BMI. Furthermore, focusing on university athletes provides valuable insight into a population that faces unique challenges in balancing academic responsibilities with athletic training demands. Overall, the findings may support the development of evidence-based nutrition education strategies aimed at improving both knowledge and practical dietary behaviors among student-athletes.

## Data Availability

The raw data supporting the conclusions of this article will be made available by the authors, without undue reservation.
